# Chemotherapeutic Drugs and Mitochondrial Dysfunction: Focus on Doxorubicin, Trastuzumab, and Sunitinib

**DOI:** 10.1155/2018/7582730

**Published:** 2018-03-18

**Authors:** Stefania Gorini, Antonella De Angelis, Liberato Berrino, Natalia Malara, Giuseppe Rosano, Elisabetta Ferraro

**Affiliations:** ^1^Laboratory of Pathophysiology of Cachexia and Metabolism of Skeletal Muscle, IRCCS San Raffaele Pisana, 00166 Rome, Italy; ^2^Department of Experimental Medicine, University of Campania “Luigi Vanvitelli”, 80138 Naples, Italy; ^3^Bionem Laboratory, Department of Experimental and Clinical Medicine, Magna Graecia University, 88100 Catanzaro, Italy; ^4^Cardiovascular and Cell Sciences Institute, St George's, University of London, Cranmer Terrace, London, UK

## Abstract

Many cancer therapies produce toxic side effects whose molecular mechanisms await full elucidation. The most feared and studied side effect of chemotherapeutic drugs is cardiotoxicity. Also, skeletal muscle physiology impairment has been recorded after many chemotherapeutical treatments. However, only doxorubicin has been extensively studied for its side effects on skeletal muscle. Chemotherapeutic-induced adverse side effects are, in many cases, mediated by mitochondrial damage. In particular, trastuzumab and sunitinib toxicity is mainly associated with mitochondria impairment and is mostly reversible. Vice versa, doxorubicin-induced toxicity not only includes mitochondria damage but can also lead to a more robust and extensive cell injury which is often irreversible and lethal. Drugs interfering with mitochondrial functionality determine the depletion of ATP reservoirs and lead to subsequent reversible contractile dysfunction. Mitochondrial damage includes the impairment of the respiratory chain and the loss of mitochondrial membrane potential with subsequent disruption of cellular energetic. In a context of increased stress, AMPK has a key role in maintaining energy homeostasis, and inhibition of the AMPK pathway is one of the proposed mechanisms possibly mediating mitochondrial toxicity due to chemotherapeutics. Therapies targeting and protecting cell metabolism and energy management might be useful tools in protecting muscular tissues against the toxicity induced by chemotherapeutic drugs.

## 1. Introduction

Many cancer therapies are known to have adverse effects. Classic chemotherapeutic cytotoxic agents as well as monoclonal antibodies against tyrosine kinase receptors, tyrosine kinase inhibitors, and antiangiogenic drugs exert cardiotoxic effects and impair the cardiovascular system by enhancing thrombotic events and by altering the hemodynamic flow. An obvious explanation for the cardiotoxicity induced by many cancer therapies is that they do not only target the tumor but also target its microenvironment. In fact, signaling pathways promoting cancer cell proliferation also protect cardiomyocytes and endothelial cells, to give two examples. Valid approaches for avoiding cancer therapy-induced cardiotoxicity need to exploit tissue-specific differences between cancer cells and the other cell types in order to target cardiotoxic mechanisms without altering the antitumor activity.

Mitochondrial dysfunctions play a prominent role in the pathogenesis of several diseases and also the cardiotoxic side effects of various drugs are often mediated by mitochondrial damage [1]. Cardiomyocytes utilize an enormous amount of ATP, therefore being in a constant energy-consuming contractile state. Since mitochondria are the ATP-producer organelles, damaged mitochondria are continuously replaced by newly synthesized ones in order to sustain the constant need for ATP. This replacement is due to processes including mitochondrial biogenesis as well as their degradation by mitophagy. These processes work in a tightly regulated manner and mitochondrial fusion and fission are regulated to create a dynamic mitochondrial network. Drugs interfering with mitochondrial functionality likely determine the depletion of ATP reservoirs and, eventually, lead to subsequent myocardial dysfunction. Mitochondrial damage may be induced in many different ways: by impairing the respiratory chain, the Krebs cycle, the oxidative phosphorylation, as well as the fatty acid *β*-oxidation. They can also depend on the loss of the mitochondrial membrane potential, on the increased oxidative stress and on the reduced antioxidative capacity. Moreover, the mitochondrial DNA (mtDNA) is proximal to the respiratory chain where most of the oxidative stress is produced. Since the mtDNA lacks both histones and repair pathways, its vulnerability to the oxidative stress has been suggested to be higher compared to nuclear DNA. mtDNA oxidation damage is cumulative and might be a major contributor to heart failure development [2–4]. Cardiac abnormalities induced via these mechanisms include cardiomyopathy, myocarditis, coronary heart disease, arrhythmias, heart failure, and Takotsubo syndrome.

Cardiac function impairment could determine, by itself, a general worsening in health, while mitochondrial dysfunctions may induce abnormalities in different body districts. Notably, skeletal muscle weakness combined with persistent fatigue is a frequent side effect in chemotherapy-treated cancer patients. The effects of chemotherapy on skeletal muscle have been found to persist for many years after treatment is discontinued and have proven to be independent on the abnormalities induced by cancer and leading to cachexia [5–8]. Due to the high metabolic expense of the skeletal muscle, the number of mitochondria is extremely high in this tissue, although lower than in cardiomyocytes [9]. Mitochondrial toxicity and dysfunctions can determine skeletal muscle-specific symptoms including weakness, atrophy, insulin resistance, impaired regenerative capacity, and exercise intolerance [10–12]. This suggests that chemotherapy could induce skeletal muscle adverse side effects, in particular, by targeting mitochondria, energy production, muscle physiology, and muscle mass [13–16].

The adverse effects of chemotherapy on skeletal muscle is more evident when therapy is administered during childhood. There is evidence that survivors of some cancers and chemotherapy-treated during childhood have high rates of skeletal muscle mass loss and dysfunction along with cardiovascular disease, insulin resistance, and metabolic syndrome several years after the treatment [8, 16, 17]. Skeletal muscle wasting affects the functional capacity of individuals but also their metabolic health. Indeed, skeletal muscle has not only contractile functions but also metabolic ones, including being a principal site of lipid oxidation and glucose uptake and, thus, a major determinant of insulin sensitivity. It has been proposed that long-term skeletal muscle dysfunction and atrophy might be preceded by mitochondrial reactive oxygen species (mtROS) production, mtDNA mutations detrimental on skeletal muscle structure and function and lasting the all life, mitochondrial impairment, and altered Ca^2+^ handling. These factors, along with DNA damaging-dependent impairment of muscle satellite cell replication and regenerative mechanisms, muscle denervation, and neuromuscular junction damage, all lead to muscle mass loss [8, 16, 18–20]. In postmitotic muscle fibers, mutated mtDNA can persist and accumulate; however, the impact of such mutations might take many years to become evident through mtDNA replication [16].

In this review, we will focus on three commonly used chemotherapeutical agents eliciting cardiotoxicity: the anthracycline doxorubicin, the erythroblastic leukemia viral oncogene homolog 2 (ErbB2) inhibitor trastuzumab, and the tyrosine kinase receptor inhibitor sunitinib. We will consider the effect of these compounds on mitochondrial activity and their effects on skeletal muscle and discuss some potential protective therapies against their adverse effects.

## 2. Doxorubicin

Anthracyclines, including doxorubicin, daunorubicin, and epirubicin, are the best studied class of anticancer agents having toxic side effects [21]. In particular, doxorubicin, discovered in the late 1960s and isolated from a culture of *Streptomyces peucetius*, is a potent anticancer agent and a treatment of first choice for many cancers, for example, the breast, liver, colon cancer, lymphoma, and leukemia [22]. However, its use is limited by a dose-dependent toxicity in many organs (e.g., the heart, brain, liver, kidney, lung, skeleton, and skeletal muscle) [23, 24]. Among others, cardiac toxicity leading to cardiomyopathy is the most serious and feared side effect of this anthracycline [23, 25, 26]; nevertheless, doxorubicin is still widely used.

**Figure 1:**
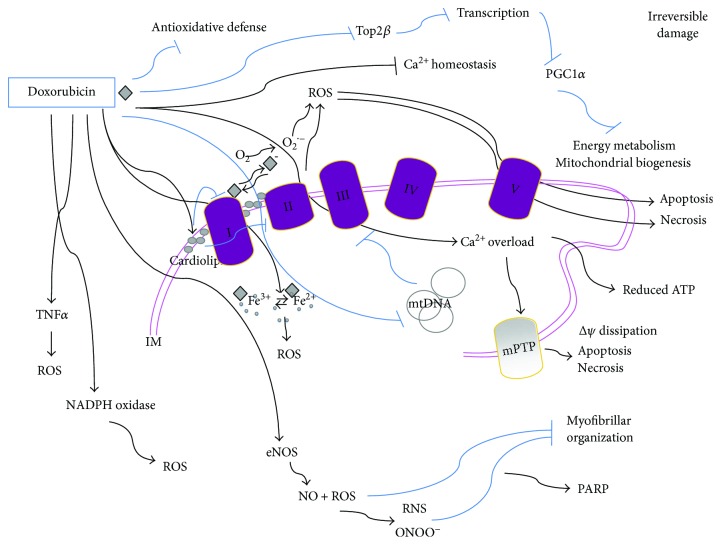
Doxorubicin-mediated cytotoxicity is mostly irreversible. Mitochondrial doxorubicin accumulation is due to its specific binding to the phospholipid cardiolipin; this membrane perturbation inhibits complex I and complex II disrupting the electron transport chain and inducing ROS production. ROS might also be produced by other doxorubicin-mediated mechanisms: a quinone moiety in the chemical structure of doxorubicin is reduced by complex I into a reactive semiquinone free radical which transfers an electron to O_2_ and generates the superoxide anion O_2_^•−^. In turn, the semiquinone free radical is oxidized and returns to the quinone form in a sequence of reactions known as the “redox cycling” of doxorubicin. Moreover, doxorubicin can directly interact with iron to form reactive anthracycline-iron complexes resulting in an iron cycling between Fe^3+^ and Fe^2+^ associated with ROS production and altering iron homeostasis. Doxorubicin also induces mtDNA damage and binds to eNOS enhancing its activity thus leading to NO production and contributing to peroxynitrite (ONOO^−^) formation. It also disrupts Ca^2+^ homeostasis which triggers mPTP and dissipates the transmembrane potential (ΔΨ) along with increasing mitochondrial permeability to apoptotic factors such as cytochrome *c* and leading to apoptosis or necrosis. The excessive oxidative stress produced by doxorubicin can also be mediated by increasing levels of TNF*α* and by NADPH oxidase and leads to redox modifications of macromolecules such as myofibrillar proteins. Doxorubicin also reduces the antioxidative defense of cells, and by preventing Top2*β* activity, it alters the transcriptome, for example, downregulating PGC-1*α*, which negatively impacts on both oxidative phosphorylation and mitochondrial biogenesis. IM: inner membrane space.

Although cardiomyocyte is the most studied and elective cellular target of doxorubicin, other cell types have been proposed as additional potential targets, making the pathogenesis of anthracycline cardiomyopathy even more complex [27]. The molecular mechanism of doxorubicin-induced cardiotoxicity is controversial. Since most cardiomyocytes are terminally differentiated cells, doxorubicin toxicity might not be only related to its anticancer effects which impair mostly DNA replication along with RNA transcription due to doxorubicin DNA intercalation and inhibition of topoisomerase II [10, 28], thus leading to cell growth and division inhibition [8]. A major hypothesis to explain doxorubicin cardiotoxicity, which is related to its cardiac accumulation and to bioactivation to secondary metabolites, involves the induction of mitochondrial abnormalities at different levels (Figure 1).

Doxorubicin specifically binds the abundant phospholipid cardiolipin located in the inner mitochondrial membrane, which leads to mitochondrial accumulation of the drug [29]. This would disrupt the electron transport chain (ETC) by inhibiting complexes I and II [30, 31] and would lead, in turn, to ROS production (Figure 1) [29, 32–35]. Indeed, doxorubicin-induced toxicity seems to be mostly due to mitochondrial increase of ROS and reactive nitrogen species (RNS), which have been proposed to be generated by “redox cycling” reactions of doxorubicin with complex I, this promoting the production of superoxide anion (O_2_^•−^) [36, 37]. More specifically, a quinone moiety in the chemical structure of doxorubicin is reduced by the respiratory chain complex I (accepting electrons from NADH or NADPH and transferring them to doxorubicin) into a reactive semiquinone free radical via one-electron reduction [36, 38]. This event removes electron normally used for ATP production and decreases the electron flow through the ETC. In normoxic conditions, the formed semiquinone transfers an electron to O_2_ and generates the superoxide anion O_2_^•−^ while being oxidized to a stable quinone in a sequence of reactions known as the “redox cycling” in which doxorubicin returns to the quinone form and the cycle continues as long as NADH is present (Figure 1). O_2_^•−^ might be transformed into the low-toxic hydrogen peroxide (H_2_O_2_) by superoxide dismutase (SOD) or into other ROS [39, 40].

Doxorubicin can also directly interact with iron to form reactive anthracycline-iron complexes resulting in an iron cycling between Fe^3+^ and Fe^2+^ associated with ROS production—including the high-toxic hydroxyl radical (OH^•^)—by the Fenton and Haber-Weiss reactions, thus altering iron homeostasis [25, 41]. Therefore, the intramitochondrial accumulation of iron is detrimental in presence of doxorubicin and is caused by doxorubicin and its metabolites, as well [25]. It is also conceivable that a secondary source of oxidants activated by doxorubicin includes NADPH oxidase. Doxorubicin also increases endothelial nitric oxide synthase (eNOS) activity and expression and, by a direct binding to this enzyme, leads to nitric oxide (NO) production and contributes to peroxynitrite formation [37, 42] (Figure 1). In line with this, the cardiomyocyte-specific overexpression of eNOS has been found to enhance the detrimental effects of doxorubicin on the heart, while eNOS-KO mice show low levels of ROS and preserved myocardial function after exposure to doxorubicin.

The increased production of mitochondrial ROS and RNS induced by doxorubicin leads to an excessive oxidative stress and is strongly linked to cell damage involving reduced protein synthesis and redox modifications of macromolecules (proteins, lipids, and DNA) such as nitrotyrosine formation, protein carbonylation, and lipid peroxidation which increase in doxorubicin-exposed cardiac muscle and include cellular membrane damage [43]. This oxidative damage results in production of stable and highly toxic aldehydes which further attack macromolecular targets. Oxidative modifications of myofibrillar proteins, such as troponin I, tropomyosin, and actin, impair maximal contraction force and compromise cardiac function [32, 33, 39, 44]. Doxorubicin-induced mitochondrial damage includes mitochondrial respiratory capacity impairment and alteration of the levels of proteins crucial for oxidative phosphorylation, as well as glycolysis and fatty acid *β*-oxidation reduction, so leading to energy substrate shifts and defective energy signaling [39] (Figure 1). Impairment of carnitine palmitoyl transferase I and *β*-oxidation is not followed by increased glucose utilization as a compensatory response. Moreover, due to its circular and covalently closed nature, mtDNA allows easy access to intercalating agents such as doxorubicin which forms adducts with mtDNA, damaging and oxidating it, thus inducing mtDNA depletion or increased rate of transcriptional errors both leading to mitochondrial dysfunctions [45]. Along with defective mitochondrial respiratory enzyme activity and increased ROS production, mtDNA damage and mutations also accumulate with repeated doxorubicin treatments [2, 46] (Figure 1).

Some authors have proposed that doxorubicin-triggered production of ROS and subsequent contractile dysfunctions might be mediated by increasing levels of tumor necrosis actor *α* (TNF*α*) [47] (Figure 1). Along with increasing oxidative stress, doxorubicin also reduces the antioxidative defense of the cell, for example, reducing glutathione (GSH), SOD, and catalase content or activity, this contributing to enhance, prolong, and stabilize the mitochondrial damage [48, 49]. In fact, doxorubicin elicits a cumulative, dose-dependent, and largely irreversible cardiac damage characterized by both structural and functional mitochondrial abnormalities and ROS-induced apoptosis and replacement by fibrotic tissue [21, 50]. By contrast, other studies have reported an increase of antioxidant enzyme activities induced by doxorubicin, this supporting the hypothesis of a cellular attempt to adapt to doxorubicin damage [10].

It has been reported that infusion of GSH or overexpression of antioxidant enzymes—for example, manganese SOD (MnSOD), catalase, glutaredoxin 2, glutathione peroxidase (GpX), and metallothionein—as well as the antioxidants vitamin E and N-acetylcysteine (NAC) reduces doxorubicin-induced toxicity [10, 51]. In line with this, it has been shown that the antioxidant and electron carrier coenzyme Q10 prevents mtDNA deletions in cardiomyocytes, suggesting a role for ROS also in mtDNA mutations [16, 52]. On the other hand, some data show that the use of antioxidants does not sufficiently protect from cardiotoxicity due to doxorubicin [25]. This is one of the reasons why some authors have proposed that the main mechanism by which doxorubicin induces cardiotoxicity is not by inducing oxidative stress but by impairing the cellular and mitochondrial Ca^2+^ signaling and homeostasis through a mechanism not yet identified [25]. Doxorubicin inhibits the transcription of the sarcoplasmic reticulum Ca^2+^-ATPase (SERCA) and therefore reduces Ca^2+^ uptake. It also targets the ryanodine receptor (RyR2) and calsequestrin type 2 (CSQ2), thus activating Ca^2+^ release channels, so increasing citoplasmatic Ca^2+^ concentration [25, 53]. Doxorubicin binds to RyR2 and SERC2A and modifies their thiols, so disrupting Ca^2+^ signaling via multiple mechanisms [53]. Therefore, doxorubicin toxicity might also be due to increased intracellular Ca^2+^ levels which may promote ROS production and impair contractile function [54, 55]. Vice versa, oxidative stress induced by doxorubicin might disrupts intracellular and mitochondrial Ca^2+^ homeostasis. Mitochondrial Ca^2+^ overload triggers mitochondrial permeability transition pore (mPTP) resulting in the dissipation of transmembrane potential, increased permeability of the mitochondrial outer membrane to apoptotic factors such as cytochrome *c* leading to apoptosis [56], and mitochondrial swelling leading to necrosis [25]. Cardiomyocyte death, both by apoptosis and necrosis ROS-induced, is a primary mechanism for anthracycline-induced cardiomyopathy [25].

Another mechanism of action of doxorubicin indirectly impacting on mitochondria involves the main target of its anticancer effect which are topoisomerase 2*α* (Top2*α*) and its isoenzyme Top2*β* which is expressed in cardiomyocytes. By preventing the Top2*β* activity, doxorubicin alters the transcriptome and downregulates the peroxisome proliferator-activated receptor-*γ* (PPAR*γ*) coactivator-1*α* and *β* (PGC-1*α* and *β*), thus impairing oxidative phosphorylation and mitochondrial biogenesis and contributing to metabolic failure. Notably, SIRT1, via PGC-1*α* deacetylation, has been implicated in the regulation of mitochondrial biogenesis. In this regard, the protective effects of SIRT1-activation in a model of anthracycline cardiotoxicity, mainly attributed to the reduction of oxidative stress and cell death, might likely also involve SIRT-1 action on mitochondrial biology and cell energetics [57, 58]. It has also been proposed that doxorubicin may additionally and indirectly act on mitochondria by acting on mitochondria-interacting desmin [49].

Finally, upregulation of apoptotic proteins and cell death is typical of doxorubicin-induced ROS-mediated cardiotoxicity [59]. Moreover, damaging the DNA, ROS, and RNS also determines the activation of the nuclear enzyme poly-ADP-ribose polymerase-1 (PARP-1) that responds to DNA damage by inducing repair using energy cofactors such as NAD^+^ [60, 61]. This determines depletion in the NAD^+^ pools and, as a consequence, in ATP stores which finally leads to inner mitochondrial membrane potential (ΔΨ) depletion and opening of mPTP, thus leading to energy homeostasis perturbation, mitochondrial swelling, outer membrane rupture, and also release of apoptotic mediators propagating the apoptotic signaling [61]. Moreover, glycolysis and tricarboxylic acid cycle (TCA), some steps of which depend on NAD^+^ availability, are also impaired by NAD^+^ depletion; as a consequence, substrate delivery to ETC and ATP synthesis is reduced.

### 2.1. Doxorubicin in Skeletal Muscle

Patients exposed to doxorubicin experience muscle weakness not relieved by rest (e.g., a slower chair-rise time and a decreased hand-grip force) up to five years following the cessation of therapy, and similarly, doxorubicin administration to rodents has been demonstrated to reduce their muscle strength [10, 47, 62]. Doxorubicin-associated skeletal muscle wasting may occur secondary to vascular dysfunction and reduced blood flow to skeletal muscles caused by doxorubicin-derived cardiac dysfunctions. However, despite the lower amount of studies on skeletal muscle, it has clearly been proven that doxorubicin directly interacts and damages skeletal muscle, reducing its strength in a dose-dependent fashion [19, 36, 63, 64].

Also in skeletal muscle, doxorubicin accumulates preferentially into mitochondria, by binding to cardiolipin. Interestingly, it has been found that a higher accumulation of doxorubicin occurs in oxidative skeletal muscles compared with the glycolytic ones [65], although this has recently been questioned [65, 66]. This might partially be explained with the higher mitochondrial mass typical of oxidative muscles. However, this cannot be the only explanation since it has been shown that doxorubicin accumulates in higher amounts in some mitochondria while being undetectable in others, possibly due to the different membrane lipidic composition of differently located mitochondria into the myofiber. Moreover, the muscle damage cannot be fully associated to the accumulation of doxorubicin also because doxorubicin metabolites (such as doxorubicinol) are more toxic than doxorubicin itself [65, 66].

Since mitochondrial density is high in skeletal muscle, it is not surprising that doxorubicin-induced mitochondrial toxicity can lead to skeletal muscle-specific symptomatology including muscle wasting, impaired regenerative capacities, and exercise intolerance [7, 10–12]. It has been shown that direct injection of doxorubicin induces skeletal muscle mass decrease and alters myofilament structure in mammals including humans, as reviewed by Gilliam and St. Clair [10]. Also, systemic doxorubicin treatment disrupts skeletal muscle myofibrillar organization and function. Similarly to myocardium, this is thought to mainly occur through disruption of redox signaling and oxidative stress induction [10, 47, 67]. In fact, circulating markers of oxidative stress, such as lipid peroxidation and protein carbonyl content, are elevated in doxorubicin-treated cancer patients. These markers might also include skeletal muscle-derived oxidants, although specific markers for skeletal muscle are not available [10]. Besides inducing protein oxidation altering myofilament structure, doxorubicin impairs mitochondrial proteins, extensively affecting muscle contractile function [10, 44, 62, 68–71]. Moreover, in skeletal muscle, similarly to myocardium, doxorubicin-induced ROS might occur also via increased levels of TNF*α* [47, 72].

Doxorubicin alters mitochondrial respiration with a subsequent increase in H_2_O_2_ emission and muscle damage. Transgenic overexpression of catalase in muscle cells blunts H_2_O_2_ emission and protein oxidation, hence protecting mitochondria as well as global muscular function. This confirms the hypothesis that mitochondrial oxidants are mediators of doxorubicin-induced skeletal muscle dysfunction [60]. In line with this, the cell-permeable peptide Bendavia (SS31), localizing to mitochondria and able to reduce ROS production, can inhibit doxorubicin-induced oxidants production in C2C12 myotubes [64, 73]. Doxorubicin treatment of myofibers has been found to lead to decreased respiratory activity, both NADH (complex I) supported and FADH_2_ (complex II) supported, along with a quick increase of H_2_O_2_ production [74]. The same authors showed that while the respiratory chain impairment remains constant, the ΔΨ, as well as the production of H_2_O_2_, decreases after a longer time of doxorubicin exposure, this indicating a decline of the overall mitochondrial function with increased sensitivity to mPTP opening and collapse of the proton motive force. More in general, doxorubicin leads to inability to maintain energy homeostasis; in fact, following doxorubicin administration, in addition to inhibition of the ETC, energy expenditure decreases, thus suggesting an impairment of the overall basal oxidative metabolism and of energy stores, for example, the activity of creatine kinase, a key enzyme for the balance of energy metabolites, decreases. Mitochondrial dysfunction might also be caused by electron leakage from the respiratory chain due to respiratory protein alterations caused by mtDNA mutations [52]. Indeed, mtDNA deletions increase with doxorubicin depending on its dosage and exposure time and by progressive amplification of mtDNA mutation, although some authors reported that skeletal muscle mass depletion by doxorubicin was associated to ROS production and respiration impairment but not to mtDNA mutation [8].

Along with enhanced ROS production, doxorubicin depletes crucial redox buffering systems, such as GSH [10, 72, 75]. Administered NAC has been found, by some authors, to be beneficial to skeletal muscle dysfunctions caused by chemotherapy [76, 77]. However, as discussed above, the effect of antioxidants is controversial also on skeletal muscle; in fact, some authors reported that the mitochondrial damage and cardiotoxicity due to doxorubcin were not reversed or prevented by NAC [78–80].

Similarly to myocardium, the DNA breaks induced by doxorubicin determine the activation of repairing enzymes among which PARPs which use energy cofactors, in particular NAD^+^, thus increasing ATP consumption and being detrimental and energetically expensive for the skeletal muscle [16, 61, 81–83]. Notably, PARP activation also reduces SIRT1 activity, mitochondrial biogenesis, and glucose metabolism, while shifting skeletal muscle fibers from the oxidative type to the glycolytic one, further promoting skeletal muscle metabolic dysfunction [16, 61, 83, 84]. In addition, a truncated form of PARP-1 has been found to act on mitochondrial proteins, this reducing mitochondrial respiration. PARP-1 pharmacological inhibition might possibly protect from NAD^+^ depletion and from metabolic impairment during chemotherapy.

High production of ROS due to doxorubicin triggers death pathways and apoptosis within skeletal muscle, involving calpain and caspase-3 activation which act also on myofilament proteins, such as the giant myofilament protein titin, this leading to sarcomere disorganization and contractile dysfunction [73, 85–87]. Doxorubicin also increases the proteasomal catabolism and induces skeletal muscle mass loss by upregulating the E3 ubiquitin ligase atrogin-1 and the ubiquitin–proteasome system [16, 64, 69, 73, 88]. Moreover, it has been suggested that damaged mitochondria might also be removed by mitophagy, possibly contributing to skeletal muscle atrophy [16].

Finally, doxorubicin reduces skeletal muscle mass also by affecting Ca^2+^ homeostasis through impairment of SERCA function and decreased Ca^2+^ uptake with increased susceptibility of the mPTP to Ca^2+^-induced opening. Doxorubicin might act like caffeine, also sensitizing the RyR2 and stimulating Ca^2+^ release from the endoplasmic reticulum [18, 53, 89, 90].

Although skeletal muscle atrophy is a common side effect of several chemotherapeutic drugs, only doxorubicin has been sufficiently studied for its direct effect on skeletal muscle [16].

## 3. Trastuzumab

Trastuzumab is a monoclonal antibody that inhibits the activation of the human epidermal growth factor receptor 2 (HER2)/neu also called ErbB2 in nonhumans, a transmembrane glycoprotein receptor with tyrosine kinase activity interfering with breast cancer growth. HER2-positive breast cancer is a highly aggressive form occurring in, approximately, one in five women [91]. The neuregulin-1/ErbB2 signaling was first recognized as having a crucial role in normal fetal heart development. Later on, the use of trastuzumab in cancer patients has highlighted the protective role of the ErbB2 signaling also on the adult heart; this signaling is essential for survival, growth, and apoptosis inhibition of cardiomyocytes [92]. In fact, the concomitant use of trastuzumab and doxorubicin was found to lead to a fivefold increase of chronic heart failure incidence in cancer patients compared to doxorubicin alone [93].

**Figure 2:**
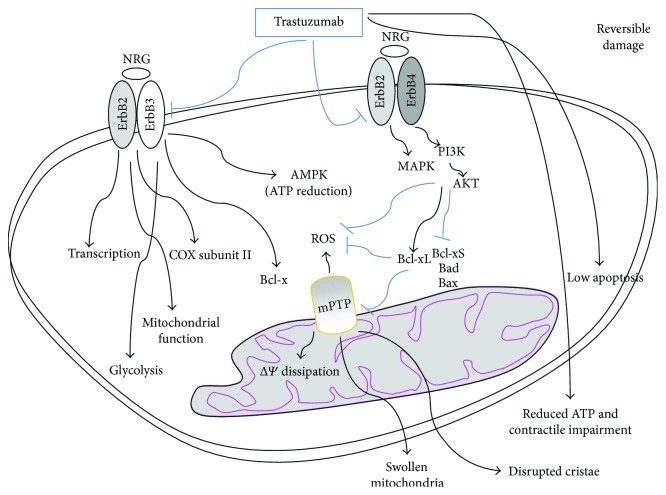
Trastuzumab-mediated cytotoxicity is mostly reversible. ErbB2 inhibition by trastuzumab reduces prosurvival signalings mediated by neuregulin-ErbB2-ErbB4 and is associated with a dramatic increase of proapoptotic Bcl-xS expression and a decrease of antiapoptotic Bcl-xL. This induces the opening of mPTP and generates ROS and mitochondrial dysfunctions among which loss of ΔΨ and ATP depletion with the disruption of cellular energetic, swollen mitochondria and reversibile contractile impairment. Cardiotoxicity of trastuzumab might also be related to its inhibition of AMPK which, in conditions of stress, leads to depletion of ATP stores. ErbB2/ErbB3 heterodimer controls Bcl-X and AMPK counteracting ATP depletion and destabilization of mitochondrial membrane, thus protecting contractile function. ErbB2 can translocate to the nucleus possibly acting on transcription and trastuzumab inhibition of ErbB2 has been suggested to regulate the expression of COX subunits.

Under biomechanical stress, hypoxia, and antracycline treatment, neuroregulin-1 secreted by the coronary endothelial cells binds to and activates ErbB4 which dimerizes with ErbB2. ErbB2/ErbB4 heterodimers promote growth and cardioprotective signaling by activating the phosphoinositide 3-kinase (PI3K), the mitogen-activated protein kinase (MAPK), and the focal adhesion kinase (FAK) survival pathways which reduce ROS production and inhibits cardiomyocyte mitochondrial apoptosis by acting on the ratio among the B-cell lymphoma 2 (Bcl-2) protein superfamily components (Figure 2) [92, 94]. By blocking the ErbB2 survival pathway, trastuzumab reduces the cancer mass along with being also severely cardiotoxic and increasing the risk of cardiovascular events whose frequency is highly variable and depends on trastuzumab association with other anticancer drugs (e.g., with or without concomitant or sequential anthracyclines), on patient age and on comorbidities [95–104].

Mice with a cardiac-specific deletion of ErbB2 develop dilated cardiomyopathy and demonstrate a robust systolic dysfunction after pressure overload compared with wild-type mice [105, 106], which confirms that the ErbB2 pathway is cardioprotective. This survival pathway is also activated following exposure to anthracyclines as a protective signaling against anthracycline-induced myocardial injury. Indeed, several clinical studies showed that, by disrupting this cardioprotective mechanism mediated by the ErbB2, trastuzumab exacerbates anthracycline-induced cardiac damage provoking an extremely high incidence of symptomatic heart failure [103]. Interestingly, ErbB2 also activates signaling molecules regulating metabolism and mitochondrial function and promotes cancer cell growth and glycolysis which are reduced by trastuzumab [107, 108]. Recent studies also have shown that ErbB2 can translocate to the nucleus, possibly acting as a transcription factor and that the expression of cytochrome *c* oxidase (COX) subunit II depends on the levels of ErbB2 expression [109, 110] (Figure 2).

By inhibiting ErbB2, trastuzumab reduces the protection from mitochondrial damage in cardiomyocytes. Trastuzumab induces cardiomyocyte toxicity through a mitochondrial pathway depending on ROS production and oxidative stress and reversed by the antioxidant NAC [111]. Indeed, cells lacking Bax and Bak which mediate cell death through a mitochondrial pathway are resistant to deleterious effects induced by trastuzumab. The mitochondrial apoptosis is regulated by the Bcl-2 protein superfamily which is also regulated by the HER2 signaling. The ratio between proapoptotic and antiapoptotic proteins determines whether the cardiomyocyte will undergo apoptosis or just reversible contractile impairment dependent on mitochondrial dysfunction. Trastuzumab activates proapoptotic proteins such as Bax and can induce the opening of mPTP, eventually leading to mitochondrial defects and dysfunctions. In fact, trastuzumab toxicity is reversed by mPTP inhibition which also reverses ROS production [111]. HER2 inhibition by trastuzumab is associated with a dramatic increase in expression of the proapoptotic Bcl-xS and decreased levels of antiapoptotic Bcl-xL [112]. These alterations induce mitochondrial dysfunctions, loss of ΔΨ, and ATP depletion with the disruption of cardiomyocyte cellular energetic and reversible contractile impairment (Figure 2).

These are the main contributors to trastuzumab cardiac toxicity associated with severe dilated cardiomyopathy but occurring with low apoptosis and alterations of the cardiomyocyte histology and ultrastructure [38, 106, 112, 113]. As a consequence, the cardiotoxic effect of trastuzumab is not cumulative or dose-related and is considered reversible, as reported in many clinical studies [50, 104, 114, 115], although this has been recently questioned [91]. Trastuzumab does not cause the typical biopsy changes observed with anthracyclines (vacuoles, necrosis, myofibrillar disarray, myocyte death) but leads to myocyte dysfunction and swollen mitochondria. Moreover, many studies have reported that the incidence of cardiac dysfunction does not increase with prolonged follow-up. In line with this, cardiomyopathy of mice with cardiac-specific deletion of HER2 might be rescued by the antiapoptotic Bcl-xL which supports the reversibility of cardiac toxicity associated with HER2 inhibition [105] by trastuzumab. Indeed, while blocking the ErbB2 signaling by trastuzumab leads to apoptosis of cancer cells [116], cardiomyocytes are particularly resistant to mitochondrial apoptosis [105, 106] possibly due to the increased expression of X-linked inhibitor of apoptosis (XIAP) and decreased expression of apoptotic protease activating factor-1 (Apaf1) [117, 118].

Based on these observations, the cardiac dysfunction induced by antineoplastic drugs such as trastuzumab has been classified for many years as type II reversible cardiotoxicity [104, 119], in opposition to the antineoplastic drugs anthracyclines leading to irreversible cardiotoxic side effects (type I cardiotoxicity). However, more recently, this classification has been questioned since an increased incidence of heart failure has been reported by some studies many years after the trastuzumab therapy, thus indicating that trastuzumab-related cardiotoxicity is not always reversible [120, 121]. On the other hand, early treatment of anthracycline-related cardiotoxic effects might, in some cases, recover cardiac function [120, 121].

Cardiomyocytes respond to biomechanical stress also by upregulating the regulator of metabolism AMPK which stimulates ATP production in order to protect the myocardium from apoptosis. ErbB2/ErbB3 heterodimer controls Bcl-X and AMPK, reducing ATP depletion and destabilization of mitochondrial membrane, thus protecting cardiomyocyte contractile function [50, 91]. It has been suggested that the cardiotoxicity of trastuzumab might also be related to its inhibition of AMPK and depletion of ATP stores [122]. By contrast, another ErbB2 inhibitor (initamib) is less cardiotoxic since it activates AMPK and increases ATP production, although not through ErbB [104]. Trastuzumab has a stronger detrimental effect on cardiomyocytes if compared with similar drugs, possibly due to its requirement of phosphatase and tensin homolog (PTEN) and of a nontruncated form of the ErbB2 receptor necessary for the ErbB2-dependent survival pathway. Finally, trastuzumab-induced cardiac toxicity has also been related to its inhibition of nuclear factor kappa beta (NF*κβ*) [104].

## 4. Sunitinib

**Figure 3:**
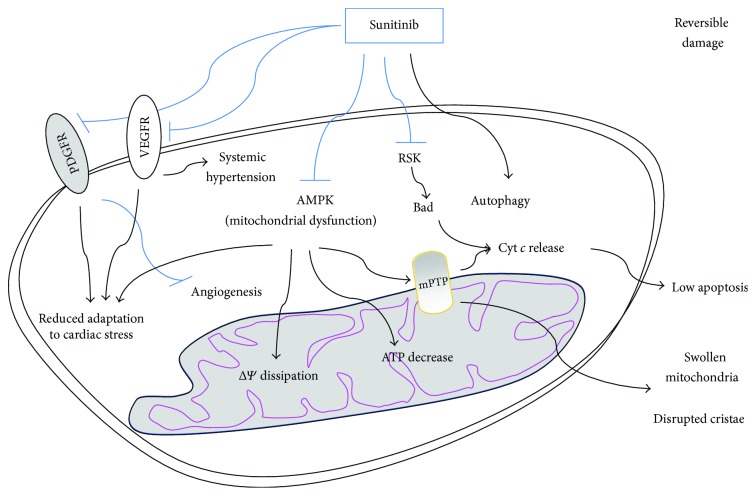
Sunitinib-mediated cytotoxicity is mostly reversible. Sunitinib impinges on cellular energy homeostasis, via disrupting the mitochondrial function through the inhibition of AMPK signaling. This induces mPTP opening, ΔΨ dissipation, swollen mitochondria, disrupted cristae, and a massive decrease of intracellular ATP, but low cytochrome *c* release and apoptosis. Sunitinib has been suggested to be also able to increase the autophagic flux and to inhibit ribosomal protein S6 kinase (RSK), thus activating Bad. By inhibiting VEGFR and PDGFR, sunitinib impairs angiogenesis and reduces adaptation to cardiac stress.

Targeted chemotherapy with tyrosine kinase inhibitors (e.g., sunitinib, lapatinib, and imatinib) is a highly selective approach which has improved the antitumor activity and the management of cancers such as renal cell carcinoma, chronic myeloid leukemia, and gastrointestinal stromal tumors along with reducing toxicities in comparison to traditional chemotherapies [123]. However, tyrosine kinase inhibitors also inhibit normal variants of these molecules in nontumor cells, which can lead to toxic side effects such as cardiotoxicity [124–126]. Sunitinib inhibits all receptor tyrosine kinases among which those for the platelet-derived growth factor (PDGF) and those for the vascular endothelial growth factor (VEGF) which are involved in angiogenesis and tumor proliferation. Sunitinib can also inhibit the AMPK pathway, as a so-called off-target toxicity. The inhibition of these pathways causes the impairment of the cardiac function [127], although the incidence of sunitinib serious adverse events is lower compared to other chemotherapeutical drugs. Sunitinib impinges on cardiac energy homeostasis by inhibiting multiple growth factor pathways (also mediated by PDGFR and VEGFR), all playing important roles in cardiomyocytes [127, 128], but also via disrupting the mitochondrial function by inhibition of the AMPK signaling, which have been extensively investigated in cultured cardiomyocytes and in mice fed with oral sunitinib (Figure 3) [127, 129]. The authors examined the endomyocardial biopsies from two gastrointestinal stromal tumors patients who had developed severe left ventricular dysfunction under sunitinib treatment; mitochondrial structure abnormalities were revealed by transmission electron microscopy. In addition, rat neonatal cardiomyocytes treated with high doses of sunitinib showed mitochondrial cytochrome *c* release and activation of caspase-9, leading to apoptosis. The exposure to sunitinib also induced ΔΨ disruption and a massive decrease in intracellular ATP [130]. Examination of cardiac tissue from mice treated daily with sunitinib revealed features similar to those observed in humans: abnormal histopathological changes including mitochondrial swelling indicating mPTP opening and energetic failure and distrupted crista. Notably, inducing hypertension in mice through administration of phenylephrine and feeding them with sunitinib showed that these animals develop a sevenfold increase in cardiac apoptosis compared to mice treated with phenylephrine alone [129]. This evidence suggests that mitochondrial dysfunction induction is the main mechanism mediating this drug's adverse effects, while cell death has been proposed to be secondary to additional cardiac impairment.

In a context of increased cardiac stress, the role of AMPK is essential in maintaining cardiac energy homeostasis; therefore, sunitinib-induced disruption of the AMPK signaling may result in the observed cardiac dysfunction [131]. AMPK acts as a master metabolic controller under conditions of metabolic stress, promoting the switch to energy generation and inhibiting anabolic pathways. AMPK regulates the activity of acetyl-CoA-carboxylase (ACC) which controls the uptake and metabolism of free fatty acids, a major source of energy in cardiomyocytes. Administration of sunitinib to animals reduces the phosphorylation of ACC in the myocardium, which indicates a reduction of AMPK activity. Under conditions of pressure overload, impaired AMPK signaling might result in failed adaptation to systolic pressure overload and might induce profound cardiac dysfunctions [132]. For this reason, the inhibition of the AMPK signaling exerted by sunitinib results in cardiac dysfunction and heart failure under increased cardiac stress conditions and may be at least partially responsible for the sunitinib-induced cardiotoxicity [130]. In fact, it has been found that overexpression of a mutant form of constitutively active AMPK protects cardiomyocytes from stress induced by sunitinib [130]. Moreover, a familial form of hypertrophic cardiomyopathy was found associate with a mutation in an AMPK regulatory subunit, which reinforces the hypothesis that AMPK signaling impairment causes heart failure, although with not fully elucidated mechanisms [133]. The AMPK activator 5-aminoimidazole-4-carboxamide-1-*β*-D-ribofuranoside (AICAR) has been studied as a potential therapy for reducing myocardial ischemic injury in both humans and animals. AMPK is a purine nucleoside analogous and is supposed to be cardioprotective acting on different pathways including reduction oxidative stress and platelet aggregation [134]. Terai and colleagues have demonstrated that AICAR attenuates cell death in rat cardiomyocytes exposed to hypoxic stress and this effect was reversed by blocking AMPK signaling [135].

Based on this data, mitochondrial dysfunction might likely be the main mechanism mediating sunitinib toxicity. Another hypothesis involves the sunitinib-dependent cytochrome *c* release and apoptosis, which might result from the activation of the proapoptotic factor Bad following the ribosomal S6 kinase (RSK) inhibition achieved by sunitinib (Figure 3) [136]. By contrast, Zhao and colleagues showed that sunitinib displays antiproliferative effect on H9c2 rat cardiac muscle cells without inducing apoptosis. In fact, all the apoptotic markers analyzed (including caspase 3 cleavage, cleaved PARP, and chromatin condensation) were not detectable after sunitinib treatment, whereas, in these cells, sunitinib dramatically increases the autophagic flux, as revealed by the high expression of microtubule-associated proteins 1A/1B-light chain 3 (LC3) II which suggests that autophagy is another sunitinib-induced process associated with its cytotoxicity [137].

We have discussed above that mitochondrial dysfunction can lead to skeletal muscle toxicity as well, including skeletal muscle atrophy, weakness, and insulin resistance. A few data are available on the effect of sunitinib on skeletal muscle. It has been found that skeletal muscle atrophy in patients with cancer is a significant predictor of dose-limiting toxicity in patients receiving sunitinib [138, 139] and that cachectic patients experience a higher sunitinib-related toxicity. Vice versa, more recently, some authors found that sunitinib prevents cachexia and muscle wasting prolonging survival of mice bearing a renal carcinoma [140]. Sunitinib was able to reverse cachectic phenotype and this was not associated to a direct antitumor activity of this drug. Although conflicting with previous data, the authors showed that skeletal muscle mass loss was also prevented in cachectic mice bearing the C26 colon carcinoma, possibly due to the ability of sunitinib to reduce the overactivation of catabolic pathways normally enhanced in cancer-induced cachexia, including STAT3 and MuRF-1 pathways [140].

## 5. Therapeutic Approaches for the Management of Chemotherapeutic Agent-Induced Damage

Among the strategies aimed at reducing the side effects of anticancer drugs [13, 141], physical activity and drugs able to protect mitochondrial metabolism are receiving increasing attention.

Exercise has been shown to mitigate doxorubicin-induced ROS production. It also protects the heart against ROS by enhancing endogenous antioxidant protective pathways [142]. Moreover, aerobic training is a potent modulator of AMPK activity in cardiac tissue and in skeletal muscle [143, 144]. Coven and colleagues have demonstrated that acute exercise increases total AMPK activity as well as the levels of the AMPK catalytic subunit isoforms and of all AMPK downstream targets [145]. Given that chemotherapeutic drugs induce downregulation of AMPK, future investigations should consider the effects of exercise on AMPK activation on cardiac and skeletal muscle function.

It has also been demonstrated that exercise is a strong activator of neuregulin release which leads to ErbB2 activation, this being a strategy to limit myocardial injury. In fact, exercise protects against Ca^2+^-induced mPTP in myocardium and reduces cell death due to doxorubicin administration, possibly by the activation of the neuregulin/ErbB2 survival pathway [146, 147]. Indeed, exercise prevents doxorubicin-induced increases of proapoptotic mediators such as Bax and caspase-3 cleavage [148].

Based on these premises, it seems conceivable that, besides exercise, molecules acting as “exercise mimetics” might also be of interest in the attempt to counteract the adverse side effects of chemotherapeutical agents. This would more likely occur when such adverse effects are mostly reversible and mainly involving mitochondrial impairment, like the ones induced by trastuzumab and sunitinib, while they might be less effective or ineffective in the case of doxorubicin-induced side effects.

Perturbation of energy homeostasis is a potent stimulus leading to skeletal muscle atrophy [11, 16]. Therefore, targeting the metabolism in order to regain the energy balance might attenuate the adverse side effects of some chemotherapeutics. We have above cited the peptide SS31, localizing to mitochondria and able to inhibit doxorubicin-induced ROS production in C2C12 myotubes [64, 73]. Also ranolazine, a member of the “metabolic modulators” group, has been found to attenuate both left ventricular diastolic and systolic dysfunction and to prevent the progression of cardiomyopathy, thus decreasing mortality, in doxorubicin-treated animals [55, 149] and in a model of cardiac cachexia [150, 151]. Notably, trimetazidine, another member of the “metabolic modulators” group, similarly to ranolazine, is able to optimize cellular energy management and has been found able to protect mitochondrial metabolism and to have effects similar to those achieved by exercise [152–154]. This drug and others with similar features are promising pharmacotherapeutics for future research in this field. Moreover, further studies are required to understand the long-term effects of anticancer treatments on skeletal muscle in order to facilitate the development of appropriate counter-measures.

## Abbreviations

ACC: Acetyl-CoA-carboxylase
ATP: Adenosine triphosphate
AMPK: 5′Adenosine monophosphate-activated protein kinase
Bak: Bcl-2-associated k protein
Bax: Bcl-2-associated x protein
Bcl2: B-cell lymphoma 2
Bcl-xS: B-cell lymphoma-extra small
Bcl-xL: B-cell lymphoma-extra large
COX: Cytochrome *c* oxidase
ErbB2: Erythroblastic leukemia viral oncogene homolog 2
eNOS: Endothelial nitric oxide synthase
ETC: Electron transport chain
FAK: Focal adhesion kinase
GpX: Glutathione peroxidase
GSH: Glutathione
HER2: Epidermal growth factor receptor 2
LC3: Microtubule-associated proteins 1A/1B-light chain 3
MAPK: Mitogen-activated protein kinase
MnSOD: Manganese superoxide dismutase
mPTP: Mitochondrial permeability transition pore
mtROS: Mitochondrial reactive oxygen species
NAC: N-Acetylcysteine
NADH: Nicotinamide adenine dinucleotide
NADPH: Nicotinamide adenine dinucleotide phosphate
NO: Nitric oxide
PARP: Poly (ADP-ribose) polymerase
PGC1*α*: Peroxisome proliferator-activated receptor gamma coactivator 1-alpha
PI3K: Phosphoinositide 3-kinase
PPAR*γ*: Peroxisome proliferator-activated receptor-*γ*
PTEN: Phosphatase and tensin homolog
RNS: Reactive nitrogen species
RyR2: Ryanodine receptor
SERCA: Sarcoplasmic reticulum Ca^2+^-ATPase
SOD: Superoxide dismutase
TCA: Tricarboxylic acid
TNF*α*: Tumor necrosis factor alpha
Top2*α*: Topoisomerase 2*α*
Top2*β*: Topoisomerase 2*β*
XIAP: X-linked inhibitor of apoptosis.
