# Corrigendum to “Chemotherapeutic Drugs and Mitochondrial Dysfunction: Focus on Doxorubicin, Trastuzumab, and Sunitinib”

**DOI:** 10.1155/2019/9601435

**Published:** 2019-06-26

**Authors:** Stefania Gorini, Antonella De Angelis, Liberato Berrino, Natalia Malara, Giuseppe Rosano, Elisabetta Ferraro

**Affiliations:** ^1^Laboratory of Pathophysiology of Cachexia and Metabolism of Skeletal Muscle, IRCCS San Raffaele Pisana, 00166 Rome, Italy; ^2^Department of Experimental Medicine, University of Campania “Luigi Vanvitelli”, 80138 Naples, Italy; ^3^Bionem Laboratory, Department of Experimental and Clinical Medicine, Magna Graecia University, 88100 Catanzaro, Italy; ^4^Cardiovascular and Cell Sciences Institute, St George's, University of London, Cranmer Terrace, London, UK

In the article titled “Chemotherapeutic Drugs and Mitochondrial Dysfunction: Focus on Doxorubicin, Trastuzumab, and Sunitinib” [1], the author Giuseppe Rosano should have been affiliated to the first and fourth affiliations. The corrected authors' list is shown above.
